# Prevalence of Diverse Genders and Sexualities in Australia and Associations With Five Forms of Child Maltreatment and Multi-type Maltreatment

**DOI:** 10.1177/10775595231226331

**Published:** 2024-01-12

**Authors:** Daryl J. Higgins, David Lawrence, Divna M. Haslam, Ben Mathews, Eva Malacova, Holly E. Erskine, David Finkelhor, Rosana Pacella, Franziska Meinck, Hannah J. Thomas, James G. Scott

**Affiliations:** 1 Institute of Child Protection Studies, 95359 Australian Catholic University, Melbourne, VIC, Australia; 2 School of Population Health, 1649 Curtin University, Perth, WA, Australia; 3 School of Law, 1969 Queensland University of Technology, Brisbane, QLD, Australia; 4 Parenting and Family Support Centre, University of Queensland, Brisbane, QLD, Australia; 5 Bloomberg School of Public Health, Johns Hopkins University, Baltimore, MD, USA; 6 56362 QIMR Berghofer, Medical Research Institute, Brisbane, QLD, Australia; 7 Queensland Centre for Mental Health Research, Wacol, QLD, Australia; 8 School of Public Health, 1974 University of Queensland, Saint Lucia, QLD, Australia; 9 Institute for Health Metrics and Evaluation, University of Washington, Seattle, WA, USA; 10 Crimes against Children Research Center, Department of Sociology, 3067 University of New Hampshire, Durham, NH, USA; 11 Institute for Lifecourse Development, 4918 University of Greenwich, London, UK; 12 School of Social & Political Science, University of Edinburgh, Edinburgh, UK; 13 Faculty of Humanities, North-West University, Vanderbijlpark, South Africa; 14 School of Public Health, University of the Witwatersrand, Johannesburg, South Africa; 15 Faculty of Medicine, 1974 The University of Queensland, Brisbane, QLD, Australia; 16 Child and Youth Mental Health Service, Children’s Health QLD, South Brisbane, QLD, Australia; 17 Child Health Research Centre, 1974 The University of Queensland, Brisbane, QLD, Australia

**Keywords:** sexual orientation, non-binary, diverse, gender, sexuality, child maltreatment

## Abstract

This study presents the most comprehensive national prevalence estimates of diverse gender and sexuality identities in Australians, and the associations with five separate types of child maltreatment and their overlap (multi-type maltreatment). Using Australian Child Maltreatment Study (ACMS) data (*N* = 8503), 9.5% of participants identified with a diverse sexuality and .9% with a diverse gender. Diverse identities were more prevalent in the youth cohort, with 17.7% of 16–24 years olds identifying with a diverse sexuality and 2.3% with a diverse gender. Gender and sexuality diversity also intersect – for example, with women (aged 16–24 and 25–44) more likely than men to identify as bisexual. The prevalence of physical abuse, sexual abuse, emotional abuse, neglect and exposure to domestic violence was very high for those with diverse sexuality and/or gender identities. Maltreatment was most prevalent for participants in the youth cohort with diverse gender identities (90.5% experiencing some form of child maltreatment; 77% multi-type maltreatment) or diverse sexualities (85.3% reporting any child maltreatment; 64.3% multi-type maltreatment). The strong association found between child maltreatment and diverse sexuality and gender identities is critical for understanding the social and mental health vulnerabilities of these groups, and informing services needed to support them.

## Introduction

Child maltreatment is the leading modifiable cause of mental and substance use disorders (Teicher et al., 2022) and its prevention is therefore a critical public health imperative globally. Although some countries such as the UK (Radford et al., 2013) and the US (Finkelhor et al., 2015) have national data about the prevalence of different types of maltreatment, few nations have conducted nationally representative surveys to establish prevalence rates of the five forms of child maltreatment—sexual abuse, physical abuse, emotional abuse, neglect, and exposure to domestic violence--experienced across childhood from age 0–18 (Mathews et al., 2020). Few look at the overlap of different advertisies, particularly the prevalence of multi-type maltreatment. Unfortunately, most epidemiological research simply reports child maltreatment prevalence data using a binary gender classification (i.e., men and women) ignoring those identifying with diverse genders.^1^ In fact, people with diverse genders are a substantially underrepresented population in research in general (Rushton et al., 2019). Similarly, attention to diverse sexuality identities has not been well addressed in prior child maltreatment prevalence estimates globally and has not featured in previous Australian prevalence assessments (Dunne et al., 2003).

Young people who identify with diverse genders or sexualities have been reported to experience higher levels of sexual violence than the general population (Ybarra et al., 2022) and are at high risk for other adversity across life including mental health disorders and bullying (Toomey & Russell, 2016). To the best of our knowledge, no Australian study has examined either the national prevalence of diverse sexuality and gender identities or the extent to which these populations report having experienced maltreatment in childhood.

### Prevalence of Diverse Genders and Sexualities in Australia

#### Gender Identity

Gender identity is distinct from sex (The Sex and Gender Sensitive Research Call to Action Group, 2020). Sex (including sex assigned at birth) is based on a person’s biological sex characteristics, such as reproductive organs or chromosomes (ABS, 2021). Sex falls into three categories: male, female and intersex. In contrast, gender identity is a social and cultural identity. Gender may differ from sex assigned at birth (Heidari et al., 2016). Common gender identities include man, woman, boy, girl, and non-binary, but also extend to transgender and gender diverse. Following established convention, we refer to persons whose gender and sex match as cisgender (McIntyre, 2018). Here, we use the term “diverse gender identities” to refer to those who are not cisgender – including trans and non-binary people (AIDS Council of New South Wales (ACON), 2019; Lyons et al., 2021).

In their cross-sectional US study of 4193 youth recruited via social media, Ybarra et al. (2022) reported that 13.9% of their self-selected participants identified as non-binary, questioning, unsure about their gender, or being of transgender experience; 7.9% identified as transgender boys or girls; and 78.3% were cisgendered. Although these are important data, recruiting participants via social media may lead to results that are not representative. As with the US, there have been no nationally representative prevalence data on gender diversity in Australia to date. The most recent national 5-yearly Census from the Australian Bureau of Statistics (ABS) conducted in 2021 did include the option to select a gender other than male or female (selected by .17%), but was widely criticised as flawed for being too general and only capturing those who identify with the specific identity of ‘non-binary’ (Lyons et al., 2021). The ABS (2022a) noted that it “cannot be used as a measure of gender diversity, non-binary genders or trans populations” (p. 1). In sum, we have little accurate representative data about population prevalence of diverse gender identities in Australia.

#### Sexuality Diversity

We use the term “diverse sexuality identities” to describe those who are not sexually attracted to the binary “opposite sex” (i.e., heterosexual). Some refer to this group as “sexual minority persons” (APA, 2021). We will include all other sexual identities or orientations in our category of ‘diverse sexuality identities’. This includes those who are same-sex attracted, as well as also those who may be attracted to all genders (i.e., pansexual), those attracted to no gender (i.e., asexual), and those with a range of other sexuality identities. Finally, we acknowledge that although gender identity and sexuality identity are distinct constructs, they obviously intersect. As noted by ACON (2019), “trans and gender diverse people have any sexual orientation including heterosexual, queer, lesbian, gay, bisexual, pansexual, asexual” (p. 2).

Although some researchers now adopt the term “sexual and gender minority (SGM)” to encompass both, the risk of using the term ‘minority’ is to encourage ‘othering’ from the heterosexual cisgender norm. Although using the concept of ‘diversity’ can still run this risk, we prefer it as a more inclusive term “sexuality and gender diverse”. People with diverse gender and/or sexuality identities may also be members of other identity groups, such as Aboriginal LGBTIQA+ people (Liddelow-Hunt et al., 2021).

Using three Australian nationally representative household surveys, namely the General Social Survey (GSS, conducted in 2014), and two waves of the Household, Income And Labour Dynamics in Australia (HILDA) Survey (2012 and 2016), Wilson et al. (2020) estimated that in Australia, 3.6% of men and 3.4% of women described themselves with a diverse sexuality identity. Interestingly, they reported important intersections between sexuality and gender identities. For example, they found that the gay population was larger than the bisexual population for men (182,100 and 77,900 respectively), but the opposite was true for women (104,400 lesbian and 137,800 bisexual). Importantly, they found a higher proportion of younger people reporting a diverse sexuality identity (Wilson et al., 2020). Although the ABS Census captured data on those living in same-sex relationships (1.4% of all couples living together in Australia in 2021), it did not measure sexuality identity (ABS, 2022b). A growing body of research about diverse sexuality and gender identities has highlighted the need for guidelines on good practice in research in this area. Heidari et al. (2016) developed a new guideline for Sex and Gender Equity in Research (SAGER) designed to ensure this research is sensitively, accurately, and consistently described – and is not confounded by other socio-economic factors. To implement this, they argued that in epidemiological studies, “the impact of other exposures, such as socio-economic variables, on health problems should be examined for all genders and should be analysed critically from a gender perspective” (p. 5). The same may be true for diverse sexuality identities.

### International Data on Gender Diversity, Sexuality Diversity, and Child Maltreatment

Violence against children exists in relation to multiple and intersecting structural and systemic forms of discrimination, such as racism, colonialism, ableism, poverty and classism, homophobia, biphobia and transphobia, and ageism that affect the vulnerability of children. However, there has been little attention documenting the prevalence of child maltreatment experiences of diverse Australians, primarily due to the lack of reliable population prevalence data about Australians who identify as having a diverse sexuality or a diverse gender identity – such as lesbian, gay, bisexual, transgender, intersex, queer, asexual and other gender/sexual minorities (LGBTIQA+) (Victorian Government, 2022). A recent review of 17 Australian studies found only 7 that included items on both gender and sexuality identity and that none of these were nationally representative (Saxby, 2022).

In the US, a recent small-scale non-representative study of sexuality diverse young adults from two urban areas showed that adverse childhood experiences (ACEs), including child maltreatment, were frequently reported – with 91.3% reporting at least one ACE, such as peer isolation/rejection (71.2%), emotional neglect (54.1%), and emotional abuse (51.1%) (Grocott et al., 2023). In their systematic review, Tobin and Delaney (2019) observed that those who display gender non-conforming traits in childhood or who are transgender are at greater risk for child abuse and adverse health and wellbeing consequences. Ybarra et al. (2022) found that compared to cisgender youths, those with diverse gender identities (i.e., transgender and non-binary) were more than twice as likely to have experienced sexual violence. Similarly, Newcomb et al. (2020) found that transgender, non-binary, and gender diverse youth were at greater risk of mental health problems, substance use, and violence. In arguing how gender and sexuality diversity can lead to adverse outcomes, Rafferty (2021) noted that

“adverse outcomes among stigmatized populations emerge through 2 forces: distal stressors from the external social environment (e.g., discrimination, stigma, abuse, and violence) and internal proximal stressors (e.g., fear of rejection, suppressing one’s identity, or internalizing negative beliefs about one’s identity)” (p. 2).

Although researchers have concluded that gender plays an important role in risk of having experienced child sexual abuse (e.g., McPhillips et al., 2022), the focus has been almost exclusively on the gender binary of man/woman. There has been less attention on gender in relation to other forms of child maltreatment, with gender minorities being largely ignored in understanding the experience, impact, treatment and prevention of child abuse and neglect.

Based on their systematic review of 32 studies of youths with sexuality diverse identities, McGeough and Sterzing (2018) concluded that compared to heterosexual youths, they experience substantially higher rates of family victimization, particularly bisexual youths. They also found that sexual minorities who experienced childhood abuse reported more frequent physical, mental, and health-related behavior problems, compared to both heterosexual or non-abused sexual minority peers who had not experienced family victimization. Although sexuality diverse youths’ experiences of family victimization were associated with adverse health outcomes, the strength of this association for youths with sexuality diversity may be different from those with diverse gender identities (McGeough & Sterzing, 2018).

In a recent cross-sectional US study of youths with data collected via social media, Mitchell et al. (2023) found that SGM youths experience disproportionately higher levels of polyvictimization, and the relationship between any individual form of victimisation and the outcomes they measured (depression; substance misuse) were attenuated after accounting for polyvictimisation. This highlights the importance of measuring and accounting for multiple forms of victimisation when trying to understand whether and how sexual/gender minority individuals are affected.

Studies on child-, parent-, and contextual-level risk factors for child maltreatment in the family typically show higher rates of child maltreatment in youths with diverse sexuality identities than for their heterosexual peers (e.g., Higgins & Hunt, 2023; Prior et al., 2021). Higher rates of physical and emotional abuse have been found to be related to disclosure of sexual orientation, younger age at first awareness of same-sex attraction, and same-sex sexual contact; and sexual and emotional abuse were associated with gender non-conformity (McGeough & Sterzing, 2018). Prior et al. (2021) found that when compared to their female peers, transgender and gender diverse groups were three times more likely to experience emotional abuse, physical abuse, sexual abuse, and emotional/physical neglect and compared to male peers, were over three times more likely to experience emotional abuse, sexual abuse, and neglect.

In a recent US study, Schnarrs et al. (2022) found that adults who identify as sexual and gender diverse commonly report childhood adversities—emotional abuse (56.1%), physical abuse (54.6%), and sexual abuse (46.2%), emotional neglect (61.6%), physical neglect (46.9%) and domestic violence (47.3%). Similarly, Jiang et al. (2023) found that 12.2% of undergraduate students in mainland China in their study self-identified as LGBQ, and that rates of childhood maltreatment were significantly higher in sexual minorities than heterosexuals. They found that emotional abuse, emotional neglect, and sexual abuse (but not physical abuse or physical neglect) were significantly associated with LGBQ identity (and with depression) in students. The likelihood, and nature of, experiencing discrimination, harassment and structural oppression—and the fear of being outed, or subjected to violence—for youths with diverse gender identities may be different to those with diverse sexuality identities, and may also intersect (for those identifying as both non-heterosexual and non-cisgendered).

Researchers have focused on the greater physical and mental disorder burdens on gender and sexuality diverse people (e.g., Lucassen et al., 2017). In a national US online study of adolescents, Thoma et al. (2021) observed that transgender adolescents reported higher rates of psychological, physical, and sexual abuse than their heterosexual cisgender counterparts. They found the greatest likelihood of having experienced psychological abuse for transgender adolescents assigned female at birth. Meyer (2003) proposed the idea of ‘minority stress’ to explain how for lesbians, gay men, and bisexuals, “stigma, prejudice, and discrimination create a hostile and stressful social environment that causes mental health problems” (p. 674). However, the reverse could also be true – that early signs of mental health problems could place gender diverse or sexuality diverse children/youth at risk for child maltreatment (see Roberts et al., 2013).

Researchers have already shown those with sexuality and/or gender diverse identities have higher risk of a range of adverse outcomes including homelessness, health risk factors (like substance use, e.g., Hughes et al., 2010) and negative mental health conditions such as depression (e.g., McNair et al., 2011; Randell & Scanlan, 2019). However, these researchers have not tested to see whether those with diverse identities in Australia differ from cisgender or heterosexual people in terms of their experiences of child maltreatment. McNair et al. (2011) highlighted the need for research to examine the social determinants of physical and mental health disparities that women with diverse sexualities experience. Child maltreatment is a major contributor to poor health, health risk behavior and poor mental health outcomes in adulthood (Lawrence et al., 2023; Pacella et al., 2023; Scott et al., 2023). Therefore, it is important to understand whether such ACEs explain at least in part the increased risk of adverse health and wellbeing outcomes.

#### The Australian Child Maltreatment Study

The Australian Child Maltreatment Study (ACMS) is the first nationally representative study of adults’ retrospective reports of all five forms of child maltreatment in Australia (Mathews et al., 2021). The ACMS provides the most rigorous population-level estimates of both gender and sexuality identities and child maltreatment, enables examination of age cohort differences (i.e., secular trends), and facilitates an exploration of any association between child maltreatment and gender/sexuality diversity. As detailed in the ACMS protocol (Mathews et al., 2021) and methodology (Haslam et al., 2023), the survey was designed to: (a) estimate the prevalence of all five types of child maltreatment (physical abuse, sexual abuse, emotional abuse, neglect, and exposure to domestic violence) in the general population; (b) determine changes over time (i.e., between different age cohorts); (c) identify associations with mental health disorders, physical health, health risk behaviors, health service utilisation, and other outcomes such as intimate partner violence in adulthood and involvement in the criminal justice system. The demographic items on self-reported gender and sexuality identity in the ACMS provide a unique opportunity to explore these issues in a population-representative sample of Australians.

### Prevalence of Child Maltreatment in Australia

Data from the ACMS are consistent with international studies showing comparably high rates of child maltreatment (Mathews, Meinck et al., 2023). Similar representative community studies show that it is considerably more prevalent than the cases of abuse and neglect that are substantiated by statutory child protection agencies (e.g., Mills et al., 2016).

Reporting on data from ACMS for the general population, Higgins et al. (2023) found 62% of the population experienced some type of maltreatment during childhood. Rates of specific types of maltreatment ranged from 8.9% (neglect) to 39.6% (exposure to domestic violence) (Mathews, Pacella et al., 2023). Single-type maltreatment was less common than multi-type maltreatment (22.8% cf. 39.4%) (Higgins et al., 2023). Child maltreatment was strongly associated with health risk behaviors (Lawrence et al., 2023), mental health disorders (Scott et al., 2023) and health service utilisation (Pacella et al., 2023). Participants with diverse gender identities had higher prevalence rates of child maltreatment compared to the full sample. This was true for all five types: physical abuse (49.9% cf. 32.0%), sexual abuse (51.9% cf. 28.5%), emotional abuse (58.3% cf. 30.9%), neglect (26.4% cf. 8.9%) and exposure to domestic violence (58.2% cf. 39.6%) (Mathews, Pacella et al., 2023) and for multi-type maltreatment (Higgins et al., 2023). Due to the comparatively low number of people identifying with diverse genders compared to those identifying as men or women, analysis of gender diversity was not incorporated into previously published data from the ACMS (Mathews, Pacella et al., 2023).

Our paper harnesses an existing nationally representative study of child maltreatment in Australia to address this gap by calculating prevalence rates of Australians identifying with diverse sexualities and/or genders, and the rates of child maltreatment across these groups. We build on the existing body of work on the prevalence of child maltreatment in Australia by specifically focusing on those Australians who identify with a diverse gender and those with diverse sexualities.

Our three broad aims were to:

1. Estimate the prevalence of people with diverse gender identities in the population in Australia, and by age cohort.
2. Estimate the prevalence of people with diverse sexuality identities in the population in Australia, and by age cohort and diverse genders; and
3. Measure the associations between gender/sexuality identities and child maltreatment (and to determine if these are influenced by socio-economic status) by estimating the prevalence of child maltreatment and multi-type child maltreatment in Australians with diverse gender identities and diverse sexuality identities.

## Method

This study used a cross-sectional retrospective interview administered via computer assisted telephone interviewing. The full methodology and sample of the ACMS has been described in detail elsewhere (see Haslam et al., 2023). Briefly, the full sample included 8503 participants, which included an over-representation of young people aged 16–24 years of age (referred to as the ‘youth cohort’) as well as 1000 participants from each decadal age cohort after (i.e., 25–34, 35–44 through to 65 years and older). For the purposes of the analyses reported here, we grouped two age deciles to create a middle-aged cohort (25–44 years) and three to create an older cohort (aged 45+). The sample was tested for representativeness against Australian Census data and national health data and received minor weightings for adjustment. The sample was also tested for evidence of potential bias towards those with a history of maltreatment and none was observed. These are detailed elsewhere (see Haslam et al., 2023).

Ethical approval was sought from the lead institution’s Human Research Ethics Committee (1900000477). Participants were recruited via random digit dialling methodology using a fully mobile sampling frame. A computer program randomly generated legitimate Australian mobile numbers based on known Australian mobile prefixes. This ensured all recruited numbers were totally random. Selected numbers received an advance text message outlining the study with a link the study Web site. Interviewers called the randomly generated numbers and invited those who answered to participate. Those who chose to participate provided verbal consent and completed the interview via phone. Distress and welfare protocols are briefly outlined in Mathews et al. (2021).

### Measures

We developed items to measure gender and sexuality identities after consultation with experts, closely aligned to the new standards outlined by the Australian Bureau of Statistics (ABS, 2021), which is responsible for the 10-yearly national census, and a range of other key government surveys and national data collections. However, ACMS items provide substantially more response options than the ABS standard against which interviewers could code responses. Although we have treated people who identify with diverse genders and those who identify with diverse sexualities as fully distinct for all analyses, we use the term “diverse gender and sexuality identities” for ease of communication when describing both populations.

To obtain participants’ gender identity, interviewers asked: “How would you describe your gender?” This was deliberately an open-response question to avoid bias. Interviewers coded from a set of response codes including male/cisgender man, female/cisgender woman, trans woman, trans man, trans femme, transmasculine, gender queer, gender diverse, non-binary, sister girl, brother boy, agender, or “I prefer not to have a label”. Any responses that could not be categorised were recorded verbatim and later coded by hand based on the closest fit to the response options. If someone responded as being a ‘man’ or a ‘woman’, we did not ask if they were cisgender or their sex assigned at birth. Therefore some non-cisgendered men/women may be included in the categories of ‘man’ or ‘woman’. However, if someone reported they were ‘cisgender’, interviewers asked them to confirm if they were a man or woman. Given the concepts of sex and gender are often—albeit incorrectly—viewed as interchangeable, we categorised anyone who responded saying ‘male’ as a man, and those responding saying ‘female’ as a woman.

To obtain participants’ sexuality identity, interviewers asked: “How would you describe your sexuality?” Response codes included heterosexual or straight, gay or lesbian, bisexual, queer, asexual, pansexual, ‘I prefer not to have a label’, and other (recorded verbatim).

The ACMS used the *Juvenile Victimization Questionnaire – R2: Adapted Version (ACMS)* (Mathews, Meinck et al., 2023) to assess experiences of child maltreatment. This included 17 screener items (yes/no response option) across five key domains of maltreatment: physical abuse (2 items, e.g., Did an adult ever hit, punch, kick or physically hurt you?), sexual abuse (5 items, e.g., Did anyone ever look at your private parts when they shouldn’t have or make you look at theirs?), emotional abuse (3 items, e.g., Did your parents often ignore you, or not show you love and affection?), neglect (3 items, e.g., Were you ever not provided with regular meals, baths or showers or clean clothes?), and exposure to domestic violence (4 items, e.g., Did you ever see or hear one of your parents get pushed, hit, choked, or beaten up by your other parent or their partner?). All items have previously been reported and show good psychometric properties (Haslam et al., 2023; Mathews, Pacella et al., 2023). The measure used behaviorally specific questions that did not require respondents to self-identify with a victim category, but simply to answer yes or no to whether during childhood they had experienced the relevant behavior. For emotional abuse and neglect, only experiences that lasted more than a week counted in our prevalence estimates. Follow-up items (not analysed in the current study) provided additional details regarding the nature of the maltreatment.

Socio-economic status was assessed using the Socio-Economic Indexes for Areas (SEIFA) Index of Relative Socio-economic Advantage and Disadvantage (ABS, 2018) based on the participant’s postcode (like a US ZIP code) of residence at the time of the survey. This index, generated by the ABS ranks geographic areas in terms of relative advantage and disadvantage using Census data on a variety of socio-economic factors such as the number of low income and high-income households and the number of skilled and unskilled workers residing in an area. This index was used to split the sample into five quintiles where the lowest quintile represents the most disadvantaged and the least advantaged locations.

### Statistical Analysis

As recommended by the SAGER guidelines (Heidari et al., 2016), we conducted gender- and sexuality-sensitive analyses of population prevalence of identity types, prevalence of child maltreatment, and the association between the two. Participants were considered to identify with a diverse gender if they responded in any way other than man or woman. Participants were considered to be sexually diverse if they responded in any way other than heterosexual or straight. Participants were considered to have experienced child maltreatment if they experienced any of the five types of maltreatment prior to age 18 years (the age of adulthood in Australia). We categorised experiences of multi-type maltreatment if they had experienced more than one of the five types of child maltreatment, or severe multi-type maltreatment if they had experienced more than two of the five types (Higgins et al., 2023). We calculated weighted prevalence estimates of diverse gender and sexuality identities, and calculated prevalence of each type of maltreatment, overall maltreatment and multi-type maltreatment by diverse sexuality and gender identities.

We then tested whether associations between gender/sexuality identities and child maltreatment are influenced by socio-economic status. We calculated prevalence of diverse gender and sexuality identities. Finally, we used logistic regression to calculate odds ratios for experience of each type of child maltreatment for women and diverse genders relative to men, and for diverse sexualities relative to heterosexual or straight adjusting for SEIFA Index of Relative Socio-economic Advantage and Disadvantage.

We used weighted survey data to represent the estimated resident population of Australia aged 16 years and over as at 30 June 2021 (Haslam et al., 2023). Compared to the 2016 Australian census, the survey sample was representative of the Australian population by region, remoteness, Indigenous status, marital status, and gender (noting that only male/female gender options were used in the 2016 census). However, survey participants were more likely to be born in Australia and have higher income, socio-economic status and level of education. We also applied survey weights that accounted for the different sample sizes in age strata (the survey was designed to have a higher representation of people aged 16–24 years) and patterns of non-response as described in Haslam et al. (2023). Confidence Intervals (CIs) for all estimates are reported and were calculated using the Surveyfreq procedure in SAS Version 9.4. Tests of statistical significance (where 95% CIs do not overlap) of difference in proportions were undertaken comparing both men and women with diverse gender identities and heterosexual or straight with diverse sexuality identities. Logistic regression models were fitted, accounting for the complex survey design using the Surveylogistic procedure in SAS Version 9.4. In total, 17 participants either said they didn’t know or refused to provide their gender.^2^ These participants have been included in “all diverse genders” subtotals to highlight differences with those who know and identify either as male or female. A much larger group of participants (*n* = 124) said they either did not know or refused to provide their sexuality identity. Because of size of this group, “don’t know/refused” was treated as a separate category for sexuality identity, rather than included in subtotals.

## Results

### Prevalence of Diverse Genders in the Australian Population

Almost 1% of the sample (*n* = 126) reported a diverse gender identity (see Table 1). The vast majority of these participants were in the youth cohort, where 2.3% of the youth identified as a diverse gender category (*n* = 90). By far, the most common response category endorsed by youth participants was non-binary (*n* = 57); however, 13 identified as gender fluid, and 10 preferred not to have a label. When weighted to represent the population of Australia 16 years and over, we estimated that almost 200,000 Australians have diverse gender identities, including 65,300 young people aged 16–24 years.

**Table 1.** Estimated Prevalence of Gender Identities in Australians Aged 16 Years and Older, by Gender Identity by Age Group.

Gender Identity	Prevalence by gender identity and age group											
	16–24 Years			25–44 Years			45 Years or More			Total		
	*n*	Weighted %	95% CI	*n*	Weighted %	95% CI	*n*	Weighted %	95% CI	*n*	Weighted %	95% CI
Prevalence by gender identity and age group												
Male/Men	1748	49.1	(47.2–50.9)	992	49.7	(47.2–52.1)	1455	46.9	(44.9–48.9)	4195	48.1	(46.8–49.5)
Female/Women	1662	48.6	(46.8–50.5)	986	49.3	(46.8–51.7)	1534	52.6	(50.6–54.6)	4182	50.9	(49.5–52.3)
Non-binary	52	1.3	(.9–1.7)	5	0.2	^a^	^a^	^a^		57	0.3	(.2–.3)
Gender fluid	8	0.2	(.1–.4)	5	0.3	(.0–.5)	0	^a^		13	0.1	(.0–.2)
Gender diverse	3	^a^		0	^a^		0	^a^		3	^a^	
Gender queer	1	^a^		1	^a^		0	^a^		2	^a^	
Trans man	5	0.1	(.0–.2)	2	^a^		0	^a^		7	0.1	(.0–.1)
Transmasculine	2	^a^		2	^a^		0	^a^		4	^a^	
Sistergirl	2	^a^		1	^a^		1	^a^		4	^a^	
Trans woman	1	^a^		2	^a^		1	^a^		4	^a^	
Trans femme	0	^a^		0	^a^		1	^a^		1	^a^	
I prefer not to have a label	4	^a^		2	^a^		4	^a^		10	0.1	(.0–.2)
Other	4	^a^		0	^a^		0	^a^		4	^a^	
Don’t know/refused	8	0.3	(.1–.4)	2	^a^		7	0.3	(.1–.5)	17	0.2	(.1–.3)
All diverse genders^b^	90	2.3	(1.8–2.9)	22	1.1	(.6–1.6)	14	0.5	(.2–.8)	126	0.9	(.7–1.2)

Gender Identity	Estimated population size by gender identity and age group							
	16–24 Years		25–44 Years		45 Years or More		Total	
	Estimate	95% CI	Estimate	95% CI	Estimate	95% CI	Estimate	95% CI
Male/Men	1,380,000	(1,310,000–1,440,000)	3,610,000	(3,380,000–3,850,000)	4,950,000	(4,690,000–5,220,000)	9,950,000	(9,600,000–10,300,000)
Female/Women	1,360,000	(1,300,000–1,430,000)	3,580,000	(3,350,000–3,820,000)	5,570,000	(5,280,000–5,860,000)	10,500,000	(10,200,000–10,900,000)
Non-binary	37,200	(25,700–48,600)	15,900	(1,400–30,400)	^a^		53,100	(34,600–71,600)
All diverse genders^b^	65,300	(50,500–80,100)	78,200	(42,000–114,000)	52,700	(22,200–83,200)	196,000	(147,000–245,000)

*Note.* In their computer-assisted coding scheme, interviewers had two other terms, ‘brother boy’ and ‘Agender’ they could have used, no respondents were coded with this gender identity, and so they are not included as columns in this table.

^a^Not published as fewer than five sample participants.

^b^All diverse genders (other than men and women). Note: In their computer-assisted coding scheme, interviewers had two other terms, ‘brother boy’ and ‘Agender’ they could have used, no respondents were coded with this gender identity, and so they are not included as columns in this table.

### Prevalence of Diverse Sexuality Identities in the Australian Population

Next, we calculated the prevalence of Australians who identify with one of the diverse sexuality identity categories (Table 2). Of the population, 90.5% identified as heterosexual or straight, and 9.5% as sexuality diverse (includes those who refused to answer or didn’t know; n = 124; 1.9%). Significantly more women than men had diverse sexuality identities. As with gender diversity, there were significant age cohort differences. The highest proportion of sexuality diversity was for those aged 16–24 (17.7%). This was significantly higher than those aged 25–44 years (9.8%), which also was significantly higher than in those aged 45 years and over (7.6%).

**Table 2.** Prevalence of Sexual Identities in Australians Aged 16 Years and Older, by Age Group.

Sexual Identity	Age Group											
	16–24 Years			25–44 Years			45 Years or More			Total		
	*N*	Weighted %	95% CI	*n*	Weighted %	95% CI	*n*	Weighted %	95% CI	*n*	Weighted %	95% CI
Prevalence by sexual identity and age group												
Heterosexual or straight	2820	81.1	(79.6–82.5)	1767	88.4	(86.8–89.9)	2833	94.4	(93.5–95.4)	7420	90.5	(89.7–91.2)
Gay or lesbian	98	2.4	(1.9–3.0)	60	2.9	(2.1–3.6)	49	1.4	(.9–1.8)	207	2.1	(1.7–2.4)
Bisexual	353	10.0	(8.9–11.1)	86	4.3	(3.3–5.2)	33	1.0	(.6–1.3)	472	3.4	(2.9–3.8)
Queer	30	0.8	(.5–1.1)	13	0.5	(.2–.9)	1	a		44	0.3	(.2–.4)
Asexual	40	1.0	(.7–1.3)	6	0.3	(.0–.6)	8	0.3	(.1–.5)	54	0.4	(.2–.6)
Pansexual	72	2.0	(1.5–2.5)	20	1.0	(.5–1.5)	5	0.1	(.0–.2)	97	0.7	(.5–.9)
I prefer not to have a label	36	1.1	(.7–1.5)	12	0.6	(.2–1.0)	18	0.5	(.2–.8)	66	0.6	(.4–.8)
Other	12	0.4	(.1–.7)	3	^a^		4	^a^		19	0.2	(.1–.3)
Sexually diverse^b^	641	17.7	(16.4–19.1)	200	9.8	(8.4–11.2)	118	3.5	(2.7–4.2)	959	7.6	(7.0–8.3)
Don’t know/refused	39	1.2	(.8–1.6)	33	1.8	(1.2–2.5)	52	2.1	(1.5–2.7)	124	1.9	(1.5–2.3)

Sexual Identity	Estimated population size by gender identity and age group							
	16–24 Years		25–44 Years		45 Years or More		Total	
	Estimate	95% CI	Estimate	95% CI	Estimate	95% CI	Estimate	95% CI
Heterosexual or straight	2,270,000	(2,190,000–2,360,000)	6,430,000	(6,130,000–6,730,000)	9,990,000	(9,600,000–10,300,000)	18,700,000	(18,300,000–19,100,000)
Gay or lesbian	68,700	(53,900–83,600)	208,000	(151,000–266,000)	147,000	(99,000–195,000)	424,000	(349,000–500,000)
Bisexual	282,000	(250,000–314,000)	311,000	(240,000–382,000)	102,000	(62,000–142,000)	694,000	(607,000–781,000)
Queer	21,200	(12,900–29,500)	40,000	(17,000–62,900)	^a^		64,000	(39,000–89,000)
Asexual	27,800	(18,700–36,900)	22,300	(3,000–41,600)	32,600	(7,900–57,400)	82,700	(50,000–115,000)
Pansexual	55,700	(41,800–69,600)	73,000	(38,000–108,000)	10,800	(600–20,900)	139,000	(101,000–178,000)
I prefer not to have a label	31,300	(20,000–42,600)	43,400	(17,400–69,400)	54,500	(24,900–84,100)	129,000	(88,000–170,000)
Other	11,200	(4,000–18,300)	^a^		^a^		42,600	(13,800–71,400)
Sexually diverse^b^	498,000	(457,000–539,000)	713,000	(606,000–820,000)	366,000	(289,000–442,000)	1,580,000	(1,440,000–1,710,000)
Don’t know/refused	33,800	(21,900–45,600)	134,000	(84,000–183,000)	223,000	(155,000–291,000)	390,000	(306,000–475,000)

^a^Not published as fewer than five sample participants.

^b^Includes all sexual identities except heterosexual or straight and don’t know/refused.

The most common diverse sexuality identity reported overall was bisexual (3.4%). This held true for the young (16–24) and middle-aged (25–44) cohorts. However, in the older-age cohort (45 years and over), gay or lesbian was the most frequently sexuality diverse identity (1.4%) – just slightly (but not significantly) higher than bisexual (1.1%). In Table 2, we also provide population estimates of the number of Australians with sexuality diverse identities. We estimate that there are almost 1.6 million Australians who identify as sexuality diverse.

We also explored the intersection between sexuality identity and gender identity. However, due to small cell sizes, we collapsed gender into three categories: men, women, and diverse genders. These data are shown in Table 3. Significantly more men than women and/or gender diverse individuals identified as heterosexual. Across the entire sample, the percentage identifying as gay or lesbian was almost double for men (2.6%) compared to women (1.4%). The reverse was true for bisexual, queer and pansexual identities. For example, there were significantly more bisexuals who were gender diverse, followed by women and then by men. The rate of bisexuality for women (4.3%) was almost double that of men (2.2%). The pattern is most pronounced for the youth cohort, where 14.9% of women identified as bisexual.

**Table 3.** Prevalence of Sexual and Gender Identities in Australians 16 Years and Older, by Sexual Identity by Gender Identity and Age Group.

		16–24 Years			25–44 Years			45^Years or More			Total		
		*n*	Weighted %	95% CI	*n*	Weighted %	95% CI	*n*	Weighted %	95% CI	*n*	Weighted %	95% CI
Heterosexual or straight	Men	1552	89.6	(88.0–91.1)	902	90.9	(89.0–92.9)	1376	95.1	(93.8–96.3)	3830	92.8	(91.8–93.8)
	Women	1263	76.2	(73.9–78.5)	859	87.0	(84.7–89.3)	1448	94.1	(92.7–95.5)	3570	89.4	(88.2–90.5)
	Diverse genders	5	3.7	(.4–7.0)	6	31.3	(8.8–53.8)	9	70.7	(45.1–96.2)	20	32.7	(19.5–45.9)
Gay or lesbian	Men	56	2.6	(1.8–3.3)	43	3.8	(2.6–5.1)	29	1.8	(1.0–2.5)	128	2.6	(2.0–3.2)
	Women	32	1.8	(1.2–2.5)	16	1.8	(.9–2.7)	20	1.1	(.5–1.6)	68	1.4	(1.0–1.9)
	Diverse genders	10	13.2	(4.4–22.1)	1	^a^		0	^a^		11	6.8	(1.2–12.3)
Bisexual	Men	83	4.6	(3.5–5.6)	26	2.8	(1.6–3.9)	17	1.0	(.5–1.6)	126	2.2	(1.6–2.7)
	Women	248	14.9	(13.0–16.8)	56	5.5	(4.0–7.1)	15	0.9	(.4–1.4)	319	4.3	(3.6–4.9)
	Diverse genders	22	24.5	(14.7–34.3)	4	^a^		1	^a^		27	14.7	(7.6–21.7)
Queer	Men	5	0.2	(.0–.4)	0	^a^		0	^a^		5	0.0	(.0–.1)
	Women	13	0.7	(.3–1.2)	9	0.7	(.2–1.2)	1	^a^		23	0.4	(.2–.6)
	Diverse genders	12	12.8	(5.4–20.2)	4	^a^		0	^a^		16	11.6	(4.2–19.1)
Asexual	Men	11	0.6	(.2–.9)	3	^a^		4	^a^		18	0.4	(.1–.6)
	Women	15	0.8	(.4–1.2)	3	^a^		4	^a^		22	0.3	(.1–.5)
	Diverse genders	14	15.1	(7.4–22.8)	0	^a^		0	^a^		14	5.0	(2.1–7.9)
Pansexual	Men	14	0.7	(.3–1.2)	3	^a^		1	^a^		18	0.2	(.1–.4)
	Women	40	2.3	(1.6–3.1)	13	1.3	.6–2.1)	4	^a^		57	0.8	(.5–1.1)
	Diverse genders	18	20.8	(11.5–30.2)	4	^a^		0	^a^		22	14.6	(6.1–23.1)
I prefer not to have a label	Men	9	0.7	(.2–1.1)	3	^a^		8	0.4	(.1–.6)	20	0.4	(.2–.6)
	Women	26	1.6	(.9–2.3)	9	0.9	(.3–1.6)	10	0.7	(.2–1.1)	45	0.9	(.5–1.2)
	Diverse genders	1	^a^		0	^a^		0	^a^		1	(a)	
Other	Men	3	^a^		1	^a^		1	^a^		5	0.2	(.0–.4)
	Women	6	0.4	(.0–.7)	0	^a^		3	^a^		9	0.2	(.0–.3)
	Diverse genders	3	^a^		2	^a^		0	^a^		5	4.3	(.0–9.0)
Don’t know/refused	Men	15	0.9	(.4–1.5)	11	1.3	(.5–2.2)	19	1.3	(.7–1.9)	45	1.2	(.8–1.7)
	Women	19	1.3	(.6–1.9)	21	2.3	(1.3–3.4)	29	2.6	(1.6–3.7)	69	2.4	(1.7–3.0)
	Diverse genders	5	5.7	(.4–10.9)	1	^a^		4	^a^		10	10.0	(2.2–17.7)

^a^Not published as fewer than five sample participants.

### Association Between Child Maltreatment and Diverse Gender Identities

As shown in Table 4, the prevalence of child maltreatment experienced by women was significantly higher than men for emotional abuse, neglect, and sexual abuse. (There were no significant differences between men and women for physical abuse and exposure to domestic violence). However, for all five maltreatment types, the prevalence for the diverse gender category was significantly higher than for women, which in turn was significantly higher than for men. The pattern was evident for physical abuse and neglect, but was strongest for sexual abuse: 51.9% of participants with diverse genders having experienced sexual abuse, compared to 37.3% of women and 18.8% of men. We found a similar pattern for experiencing any type of child maltreatment, with the lowest prevalence for men (58.4%), significantly higher for women (65.5%), and significantly higher yet again for those with diverse gender identities (81.5%). It is striking that relationship between child maltreatment and diverse gender identities was evident across age groups. Across all gender identity categories, the youth cohort had considerably higher prevalence of each form of child maltreatment, and of multi-type maltreatment, than both older age cohorts (we were unable to report the exact numbers due to cell sizes being below the threshold for reporting of *n* = 5). There appears to be no significant age-group differences in the comparative risk of each and any child maltreatment by diverse gender identities.

**Table 4.** Prevalence of Child Maltreatment, by Age Group, Gender Identity and Type of Maltreatment.

		Emotional Abuse		Neglect		Physical Abuse		Sexual Abuse		Exposure to Domestic Violence		Any child Maltreatment	
		%	95% CI	%	95% CI	%	95% CI	%	95% CI	%	95% CI	%	95% CI
16–24 years	Men	26.9	(24.6–29.3)	7.2	(5.8–8.5)	26.3	(24.0–28.5)	14.5	(12.6–16.5)	40.8	(38.2–43.3)	55.5	(52.9–58.0)
	Women	40.5	(37.9–43.1)	12.5	(10.8–14.2)	29.0	(26.6–31.4)	35.2	(32.7–37.8)	45.8	(43.2–48.5)	65.5	(63.0–68.1)
	Diverse genders	**71.5**	**(61.3–81.7)**	**30.6**	**(19.9–41.2)**	**53.8**	**(42.5–65.0)**	**62.8**	**(51.8–73.8)**	**64.8**	**(54.0–75.5)**	**90.5**	**(84.0–97.0)**
25–44 years	Men	27.0	(23.9–30.1)	7.0	(5.3–8.8)	33.4	(30.2–36.7)	18.5	(15.8–21.2)	44.3	(40.9–47.8)	62.5	(59.1–65.8)
	Women	40.3	(37.0–43.7)	13.7	(11.2–16.2)	35.6	(32.3–38.9)	38.8	(35.4–42.2)	47.4	(44.0–50.8)	70.4	(67.2–73.5)
	Diverse genders	**70.3**	**(48.6–92.0)**	28.6	(7.3–49.9)	50.8	(27.9–73.7)	47.4	(24.6–70.2)	65.6	(43.1–88.2)	81.8	(61.2–100.0)
45 years or more	Men	23.8	(21.4–26.2)	6.3	(4.8–7.7)	32.8	(30.1–35.5)	20.2	(17.9–22.4)	32.6	(30.0–35.3)	56.2	(53.3–59.0)
	Women	31.4	(28.8–34.0)	8.4	(6.8–10.0)	29.5	(27.0–32.1)	36.9	(34.2–39.6)	35.2	(32.6–37.9)	62.4	(59.7–65.2)
	Diverse genders	^a^		^a^		43.7	(14.6–72.7)	45.2	(16.2–74.1)	39.0	(11.3–66.7)	70.0	(44.0–96.1)
Total	Men	25.4	(23.7–27.1)	6.7	(5.7–7.6)	32.1	(30.3–33.9)	18.8	**(17.3–20.3)**	38.0	(36.1–39.9)	58.4	(56.5–60.3)
	Women	35.6	(33.8–37.4)	10.8	(9.5–12.0)	31.5	(29.7–33.3)	37.3	(35.5–39.2)	40.8	(38.9–42.6)	65.5	(63.7–67.4)
	Diverse genders	**58.3**	**(45.6–71.1)**	**26.4**	**(15.5–37.4)**	**49.9**	**(37.3–62.5)**	**51.9**	**(39.3–64.6)**	**58.2**	**(45.5–70.9)**	**81.5**	**(70.4–92.6)**

^a^Not published as fewer than five sample participants.

Bold signifies significantly different than Men or Women *p* < .05.

A much stronger pattern was evident when individuals experienced two or more types of child maltreatment (multi-type maltreatment). Gender diverse indiviuals were significantly more likely to experience multi-type maltreatment than women, who were significantly more likely to experience it than men (see Table 5). The prevalence of severe multi-type maltreatment (three or more types) in those with a diverse gender identity was almost double (51.2%) the rate for women (27.6%), which was significantly higher than for men (18.1%). For multi-type maltreatment (2 or more types), the pattern was similar, although not quite as strong as for severe multi-type maltreatment (3 or more types). The strong pattern was driven in part by the very strong associations and signitificant differences found for the youth cohort, but was less strong for the 25–44, and 45 years and over age groups (see Table 5).

**Table 5.** Prevalence of Child Maltreatment, by Age Group, Gender Identity and Number of Types of Maltreatment Experienced.

		No Maltreatment		One Type of Maltreatment		Two or More Types of Maltreatment		Three or More Types of Maltreatment	
		%	95% CI	%	95% CI	%	95% CI	%	95% CI
16–24 years	Men	44.5	(42.0–47.1)	22.4	(20.2–24.7)	33.0	(30.6–35.5)	18.6	(16.6–20.7)
	Women	34.5	(31.9–37.0)	19.9	(17.8–22.1)	45.6	(42.9–48.3)	30.2	(27.7–32.6)
	Diverse genders	**9.5**	**(3.0–16.0)**	13.0	(6.1–20.0)	**77.5**	**(68.4–86.5)**	**66.0**	**(55.6–76.3)**
25–44 years	Men	37.5	(34.2–40.9)	24.1	(21.1–27.1)	38.4	(35.0–41.7)	19.2	(16.5–21.9)
	Women	29.6	(26.5–32.8)	21.2	(18.4–24.0)	49.2	(45.7–52.6)	31.6	(28.4–34.8)
	Diverse genders	^a^		^a^		70.3	(48.6–92.0)	**57.1**	**(34.2–79.9)**
45 years or more	Men	43.8	(41.0–46.7)	23.4	(20.9–25.8)	32.8	(30.1–35.5)	17.3	(15.1–19.4)
	Women	37.6	(34.8–40.3)	23.7	(21.3–26.1)	38.7	(36.0–41.5)	24.4	(22.0–26.8)
	Diverse genders	30.0	(3.9–56.0)	^a^		^a^		^a^	
Total	Men	41.6	(39.7–43.5)	23.5	(21.8–25.1)	34.9	(33.0–36.7)	18.1	(16.7–19.6)
	Women	34.5	(32.6–36.3)	22.4	(20.7–24.0)	43.2	(41.3–45.1)	27.6	(25.9–29.3)
	Diverse genders	**18.5**	**(7.4–29.6)**	15.4	(6.8–23.9)	**66.2**	**(53.8–78.5)**	**51.2**	**(38.6–63.8)**

^a^Not published as fewer than five sample participants.

Bold signifies significantly different than Men or Women at *p* < .05.

### Association Between Child Maltreatment and Diverse Sexuality Identities

We calculated the prevalence of each of the five domains of child maltreatment, the experience of any child maltreatment, and the experience of multi-type maltreatment, separately for each groups: heterosexual/straight, all other sexuality identity categories combined (‘sexuality diverse’), and those who refused or didn't know. As shown in Table 6, the prevalence of child maltreatment experienced by sexuality diverse Australians was significantly higher compared to heterosexuals for each type of child maltreatment, but particularly so for sexual abuse (51.9% cf. 20.1%). Overall, the trends for any type of child maltreatment followed a similar pattern, with lower prevalence for heterosexuals (61.0%), compared to diverse sexuality identities (83.9%). Significant differences were found across each age group (where we had sufficient sample size to estimate).

**Table 6.** Prevalence of Child Maltreatment, by Age Group, Sexuality Identity and Type of Maltreatment.

		Emotional Abuse		Neglect		Physical Abuse		Sexual Abuse		Exposure to Domestic Violence		Any child Maltreatment	
		%	95% CI	%	95% CI	%	95% CI	%	95% CI	%	95% CI	%	95% CI
16–24 years	Heterosexual or straight	29.6	(27.7–31.5)	7.9	(6.8–9.0)	24.9	(23.1–26.7)	20.1	(18.5–21.8)	40.7	(38.7–42.8)	56.2	(54.2–58.2)
	Sexuality diverse	**58.0**	**(53.8–62.2)**	**21.6**	**(18.1–25.2)**	**44.6**	**(40.3–48.8)**	**51.9**	**(47.6–56.2)**	**58.6**	**(54.4–62.8)**	**85.3**	**(82.3–88.2)**
	Don’t know/refused	23.4	(10.3–36.4)	^a^		**13.2**	**(3.2–23.2)**	15.0	(4.0–26.0)	31.1	(15.9–46.3)	40.2	(23.7–56.8)
25–44 years	Heterosexual or straight	32.0	(29.6–34.5)	9.4	(7.8–11.0)	33.5	(31.1–36.0)	26.4	(24.1–28.7)	45.0	(42.5–47.6)	65.2	(62.8–67.7)
	Sexuality diverse	**56.3**	**(48.8–63.8)**	**20.7**	**(14.5**–**26.9)**	**48.3**	**(40.8**–**55.9)**	**52.4**	**(44.9–60.0)**	**60.5**	**(53.1**–**67.9)**	**85.6**	**(80.1–91.1)**
	Don’t know/refused	^a^		^a^		**17.2**	**(3.7–30.7)**	17.3	(3.6–31.0)	**18.7**	**(5.7–31.7)**	**28.3**	**(12.3–44.4)**
45 years or more	Heterosexual or straight	27.1	(25.3–29.0)	7.3	(6.2–8.4)	31.0	(29.1–32.9)	28.5	(26.6–30.3)	33.6	(31.6–35.5)	59.3	(57.2–61.3)
	Sexuality diverse	**50.1**	**(39.6–60.7)**	13.5	(6.0–21.1)	**43.7**	**(33.3–54.1)**	**53.8**	**(43.3–64.3)**	**52.8**	**(42.3–63.3)**	**78.6**	**(69.6–87.6)**
	Don’t know/refused	20.2	(7.0–33.4)	^a^		**18.2**	**(6.8–29.6)**	**15.6**	**(4.0–27.1)**	24.1	(10.1–38.1)	**39.6**	**(24.7–54.5)**
Total	Heterosexual or straight	29.1	(27.8–30.4)	8.1	(7.3–8.9)	31.1	(29.8–32.4)	26.8	(25.5–28.0)	38.4	(37.0–39.8)	61.0	(59.6–62.4)
	Sexuality diverse	**55.4**	**(51.0–59.8)**	**19.3**	**(15.8–22.8)**	**46.1**	**(41.7–50.4)**	**52.6**	**(48.2–57.0)**	**58.1**	**(53.8–62.5)**	**83.9**	**(80.5–87.3)**
	Don’t know/refused	**17.6**	**(9.0–26.2)**	6.3	(1.2–11.3)	**17.4**	**(9.4–25.4)**	**16.1**	**(8.0–24.3)**	**22.9**	**(13.5–32.2)**	**35.8**	**(25.4–46.2)**

^a^Not published as fewer than five sample participants.

Bold signifies significantly different than heterosexual/straight at *p* < .05.

Again, a much stronger pattern between diverse sexualities and child maltreatment was evident when individuals experienced multi-type maltreatment. Sexuality diverse individuals were significantly less likely to experience no maltreatment, and significantly more likely to experience multi-type maltreatment (2+ or 3+ types) than heterosexuals. As shown in Table 7, the proportion of people with diverse sexualities experiencing three or more types (46.8%) was significantly higher – more than double the rate for heterosexuals (21.5%). This pattern of significantly higher multi-type maltreatment experienced by sexuality diverse individuals was fairly consistent across the youth cohort, the 25–44, and 45 years and over age groups (see Table 7). As we found with gender diversity, a similarly striking pattern is that there appears to be no significant age-group differences in the comparative ORs of each and any child maltreatment by diverse sexuality identities.

**Table 7.** Prevalence of Child Maltreatment, by Age Group, Sexual Identity and Number of Types of Maltreatment Experienced.

		No Maltreatment		One Type of Maltreatment		Two or More Types of Maltreatment		Three or More Types of Maltreatment	
		%	95% CI	%	95% CI	%	95% CI	%	95% CI
16–24 years	Heterosexual or straight	43.8	(41.8–45.8)	21.1	(19.4–22.8)	35.1	(33.1–37.1)	20.5	(18.8–22.1)
	Sexuality diverse	**14.7**	**(11.8–17.7)**	21.0	(17.6–24.4)	**64.3**	**(60.2–68.3)**	**48.1**	**(43.8–52.3)**
	Don’t know/refused	59.8	(43.2–76.3)	13.6	(2.7–24.6)	26.6	(12.5–40.7)	19.6	(7.2–32.0)
25–44 years	Heterosexual or straight	34.8	(32.3–37.2)	23.3	(21.1–25.5)	42.0	(39.4–44.5)	23.4	(21.2–25.6)
	Sexuality diverse	**14.4**	**(8.9–19.9)**	19.1	(13.2–25.0)	**66.5**	**(59.3–73.7)**	**48.2**	**(40.7–55.7)**
	Don’t know/refused	**71.7**	**(55.6–87.7)**	^a^		24.1	(8.6–39.7)	15.9	(2.6–29.3)
45 years or more	Heterosexual or straight	40.7	(38.7–42.8)	23.9	(22.1–25.6)	35.4	(33.4–37.4)	20.4	(18.8–22.1)
	Sexuality diverse	**21.4**	**(12.4–30.4)**	19.4	(11.3–27.4)	**59.2**	**(48.9–69.6)**	**42.3**	**(31.9–52.6)**
	Don’t know/refused	60.4	(45.5–75.3)	15.2	(5.6–24.8)	24.4	(10.4–38.3)	14.9	(2.6–27.2)
Total	Heterosexual or straight	39.0	(37.6–40.4)	23.3	(22.1–24.6)	37.6	(36.2–39.0)	21.5	(20.3–22.6)
	Sexuality diverse	**16.1**	**(12.7–19.5)**	19.8	(16.3–23.2)	**64.1**	**(59.9–68.4)**	**46.8**	**(42.4–51.2)**
	Don’t know/refused	**64.2**	**(53.8–74.6)**	**11.3**	**(5.4–17.2)**	**24.5**	**(14.8–34.1)**	15.7	(7.3–24.1)

^a^Not published as fewer than five sample participants.

Bold signifies significantly different than heterosexual/straight at *p* < .05.

**Figure 1.**
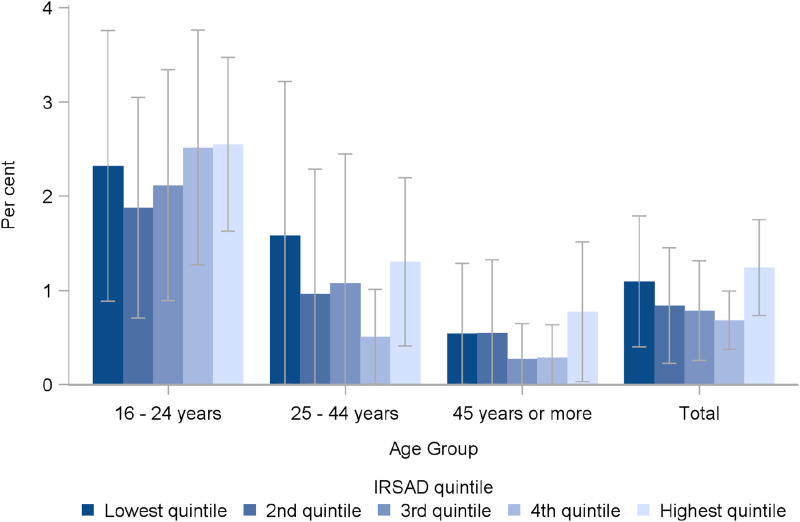
Proportion of Australians identifying as gender diverse, by age group and SEIFA index of relative socio-economic advantage and disadvantage (overlapping CIs show no significant differences).

**Figure 2.**
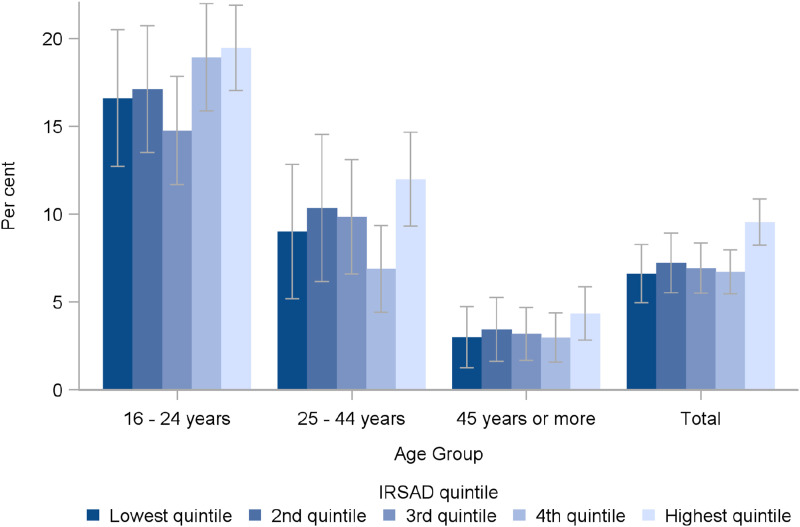
Proportion of Australians identifying as sexuality diverse, by age group and SEIFA index of relative socio-economic advantage and disadvantage (overlapping CIs show no significant differences).

As recommended by the SAGER guidelines, we then explored whether there was any relationship between socio-economic status (using the SEIFA index) and gender and sexuality diversity. We found no significant differences in prevalence of diverse gender (Figure 1) or sexuality (Figure 2) identities by quintiles of the SEIFA Index of Relative Socio-economic Advantage and Disadvantage. We also calculated ORs for experience of child maltreatment relative to men (for diverse gender identities), or to heterosexuals (for diverse sexuality identities). Using logistic regression models adjusting for age group and SEIFA, we concluded that all of the ORs for increased risk of child maltreatment remained significantly elevated for people with diverse genders (see Table 8) or diverse sexualities (see Table 9). While fitting the logistic regression models, we tested for interactions between gender diversity and SEIFA and between sexuality diversity and SEIFA and noted no significant interaction effects for any type of child maltreatment, suggesting that the relationship between gender diversity, sexuality diversity and child maltreatment was independent of socio-economic status.

**Table 8.** Odds Ratios (ORs) for Experience of Child Maltreatment, by Gender and Type of Maltreatment, Adjusting for Age Group and SEIFA Index of Relative Socio-Economic Advantage and Disadvantage.

	Men		Women		Diverse Genders	
	OR	95% CI	OR	95% CI	OR	95% CI
Any child maltreatment	1.00	(ref)	**1.37**	**(1.22–1.53)**	**3.08**	**(1.46–6.53)**
Emotional abuse	1.00	(ref)	**1.64**	**(1.45–1.85)**	**3.86**	**(2.29–6.50)**
Neglect	1.00	(ref)	**1.70**	**(1.38–2.08)**	**4.77**	**(2.63–8.64)**
Physical abuse	1.00	(ref)	.97	(.86–1.09)	**2.15**	**(1.28–3.62)**
Sexual abuse	1.00	(ref)	**2.57**	**(2.26–2.92)**	**4.84**	**(2.86–8.16)**
Exposure to domestic violence	1.00	(ref)	**1.14**	**(1.02–1.27)**	**2.08**	**(1.24–3.51)**

Bold signifies significantly different than Men at *p* < .05.

**Table 9.** Odds Ratios (ORs) for Experience of Child Maltreatment, by Sexuality Identity and Type of Maltreatment, Adjusting for Age Group and SEIFA Index of Relative Socio-Economic Advantage and Disadvantage.

	Heterosexual or Straight		Sexuality Diverse		Don’t Know/Refused	
	OR	95% CI	OR	95% CI	OR	95% CI
Any child maltreatment	1.00	(ref)	**3.33**	**(2.56–4.34)**	**.35**	**(.22–.55)**
Emotional abuse	1.00	(ref)	**2.90**	**(2.39–3.52)**	**.52**	**(.28–.94)**
Neglect	1.00	(ref)	**2.61**	**(2.02–3.37)**	.74	(.31–1.75)
Physical abuse	1.00	(ref)	**1.98**	**(1.63–2.40)**	**.45**	**(.26–.80)**
Sexual abuse	1.00	(ref)	**3.35**	**(2.75–4.07)**	**.51**	**(.27–.93)**
Exposure to domestic violence	1.00	(ref)	**2.02**	**(1.66–2.45)**	**.47**	**(.27–.82)**

Bold signifies significantly different than heterosexual/straight at *p* < .05.

## Discussion

This paper presents the most comprehensive nationally representative prevalence of diversity in adult Australians (in terms of both gender and sexuality identities) and the associations with childhood experiences of five separate forms of maltreatment and their intersection: multi-type maltreatment. As recommended by Heidari et al. (2016), we conducted both sexuality and gender sensitive analyses, and considered the relevance of child maltreatment prevalence research for diverse populations.

We found the prevalence of people with diverse genders is .9% in the Australian population. Our methods (random digit dial; sampling adults across the lifespan) may explain why this prevalence is considerably lower than recent US studies of youth recruited via social media, which found up to 13.9% of participants had gender diversity (Mitchell et al., 2023; Ybarra et al., 2022). Our population-representative data showed that diversity varied across the age cohorts. The much greater proportion of our youth cohort identifying with a diverse gender identity (2.3%) is likely to reflect the greater social awareness and acceptability of gender not being a binary of male/female.

For the first time, we also have population-representative data showing that it is common for people in Australia to have diverse sexuality identities (9.5%). There are important differences based on the age and gender. We observed much greater sexuality diversity in the youth cohort (17.7%), as well as gender differences in sexuality diversity (such as women more likely to identify as bisexual also intersecting with cohort differences, with 14.9% of women in the youth cohort identifying as bisexual). The differences across age cohorts are again evidence that social changes, including anti-discrimination laws, education and exposure to diversity in the media, particularly during the lead up to the 2017 postal survey (‘plebicite') where the majority of Australians agreed to change the law allowing same-sex couples to marry, are likely to have contributed to greater awareness and acknowledgement of diversity in young people in Australia compared to older cohorts.^3^

Importantly, we found that child maltreatment—and particularly the intersection of these harms: multi-type child maltreatment—was more prevalent among Australians with diverse gender identities as well as in those with diverse sexuality identities. The likelihood of having experienced child maltreatment was strongest for those with gender diversity in the youth sample, with 90.5% reporting any type of child maltreatment, and 77.5% experiencing multi-type maltreatment. The association was almost as strong for diverse sexuality identities, with 85.3% of sexually diverse participants reporting any child maltreatment (and 64.3% reporting multi-type maltreatment).

### Prevalence of Gender and Sexuality Diversity

Our population estimate for all diverse gender identities of .9% obscures a very strong secular trend, with much lower rates of diverse genders in the middle aged (1.1%) and older cohorts (.5%) compared to the youth sample, which had the highest prevalence of diverse genders (2.3%). We observed double the rate of participants with sexually diverse identities (7.6%) than Wilson et al.’s (2020) estimates for Australia of 3.6% for men and 3.4% for women. We also found a much higher proportion of younger people reporting a diverse sexuality identity than older participants, as did Wilson et al. We also noted this same pattern for diverse genders. Like Wilson et al., we reported that gender and sexuality intersect – for example, with women more likely to identify as bisexual than men. Extrapolating our sample data to the Australian population means that around 200,000 Australians identify with diverse genders, and around 1.6 million with diverse sexualities. This needs to be a major focus of prevention and response initiatives, given our findings of the significantly increased risk of multi-type child maltreatment. These numbers are likely to keep increasing, given the stark differences we identified between the youth sample and the older age cohorts.

### Diversity and Exposure to Harm

Our data, from adults aross the lifespan, align with Mitchell et al.’s 2023 study of 14–15 years olds showing that the most common experience of sexuality and gender diverse youths is to have childhoods characterised by multiple adversities. We also found significantly higher exposure to multi-type (and severe multi-type) maltreatment. The strong association between multi-type child maltreatment and both gender and sexuality diversity may help explain some of the unique health disparities and the burden of disease in terms of mental health that has been found for gender and sexually diverse people (Institute of Medicine (US) Committee on Lesbian, Gay, Bisexual, and Transgender Health Issues and Research Gaps and Opportunities, 2011). A unique finding from our study is that the associations were strong not just for experiencing a single type, but also for experiencing multi-type maltreatment. Given the evidence on the strong relationship between experiences of multi-type maltreatment and the physical and mental disorders, health-risk behaviors and health service utilization (Lawrence et al., 2023; Pacella et al., 2023; Scott et al., 2023), it is likely that the higher rates of mental and physical health problems in those who identify with diverse sexualities and genders may be explained in part by the high prevalence of multi-type maltreatment in these two groups.

Similar to North American research (e.g., Grocott et al., 2023; Tobin & Delaney, 2019), child maltreatment was strongly associated with gender diversity in the ACMS – particularly for the youth cohort. Other research from Australia has shown that young people with non-binary (diverse gender) identities are more likely to have lower perceptions of safety in institutions (Russell et al., 2020). The ACMS results may provide an explanation for Russell et al.’s finding that gender diverse young people feel less safe in institutions, due to the significantly greater likelihood of their exposure to one of the most damaging experiences of child maltreatment: multi-type maltreatment. As noted by Hamby et al. (2021), there is greater recognition now of the problem of narrowing in and focusing on one type of trauma without taking into account the likelihood of experiencing multiple adversities. Researchers and practitioners need to avoid silos, and accept that multiple advertisities are the ‘norm’ for those who experience child maltreatment, but even more so for those who are gender and/or sexuality diverse. Importantly, the greater burden of trauma for gender and sexuality diverse inidivduals, and the often-cited likelihood of greater mental health problems in these populations, is likely attributable to the cumulative effects of multiple adversities they have experienced in childhood (e.g., Randell & Scanlan, 2019).

Although diversity in gender and sexuality were both strongly associated with child maltreatment experiences, the mechanisms and causal relationships are unclear. A child or young person who presents outside of the ‘norms’ of gender or sexuality expression may make them a target for victimisation by parents, carers, other family members, peers or staff in educational and youth-serving organisations with which they engage. In relation to child sexual abuse, it may be that those perpetrating sexual abuse select victims based on vulnerability (such as being part of a sexuality and/or gender diverse identity group), due to their greater risk of social isolation, and their need for love and acceptance (and therefore susceptibility to grooming). It is unclear whether such patterns of vulnerability may also apply to those experiencing other forms of child maltreatment, or exposure to multi-type maltreatment. Children or young people who express themselves in ways that do not align with gender norms and behaviors from an early age may therefore be at risk of maltreatment from parents, carers, other adults, or victimisation from siblings or other children and young people (including sexual victimsation).

In a rare prospective longitudinal study, Xu et al. (2020) found that maternal reports of childhood paternal physical or emotional maltreatment prior to age 7 were associated with self-reported same-sex sexual orientation at age 15 for boys (but not statistically significant for girls). However, they did not explore the relationship for other forms of child maltreatment, including sexual abuse. Without comprehensive prospective data it is difficult to fully understand temporal sequencing let alone causality. Moreover, the stigma, shame, silence, and difficulty with speaking up can apply both to victimisation from child maltreatment and to coming out with a diverse gender or sexuality identity. Speaking up or coming out are both likely to have significant time lags between behaviors or events and even the earliest of internal awareness, and cognitive framing of them. Parental rejection, attempts to impose conventional behavior, sibling abuse, peer abuse or other victimisation may result from a child or young person’s (presumed or disclosed) diverse identity.

Our findings highlight the rapid increase in recent years of gender and sexuality diversity in the population in Australia, with current rates likely to be much higher than previously estimated. The significantly higher likelihood of gender and sexuality diverse Australians having experienced child maltreatment—particularly multi-type maltreatment—has important implications for policies and services. A greater climate of acceptance and recognition of diversity is needed to underpin general child maltreatment prevention and response strategies. Our estimates of the prevalence of gender and sexuality diversity, together with the significantly greater odds not only of any form of child maltreatment but of its most damaging form – when multiple types are experienced – should make this group a high priority for service providers delivering therapeutic interventions, redress schemes or other justice responses and supports for victim/survivors. Diversity groups must also be a priority focus in prevention initiatives, from child-sexual abuse safeguarding strategies in organisations, through to parenting advice and programs to support parents of children with emerging diverse identities.

#### Approaches to Prevention

Strategies to prevent or reduce risks of each form of child maltreatment need to take into account gender and sexuality diversities. It may be that existing approaches do not work as well for ensuring the safety of sexuality and/or gender diverse youths, or that additional attention and unique strategies might be needed. For example, in attempts to improve the quality of parenting for families with gender and/or sexuality diverse children and youths, this should include both guidance and supports for parents (parenting skills, and specific safeguarding advice and skills building) that are tailored to their unique circumstances. Similarly, in terms of addressing situational risks of child sexual abuse, organisational safeguarding strategies might need to be amplified and adjusted to take into account the greater vulnerability of sexuality and gender diverse young people, such as strategies to address transport, sleeping arrangements, or access to appropriate bathroom facilities while on camps, excursions or other outings.

#### Approaches to Treatment and Responses to Child Maltreatment

Clinicians must acknowledge not only the diversity of gender and sexuality identities we found in Australia, but the increased likelihood that those with diverse identities have experienced child maltreatment, particularly multi-type maltreatment. They should take into account gender and sexuality diversities in their interventions, in terms of considering whether the evidence of the effectiveness of their treatment might vary for adults who are heterosexual versus sexuality diverse, and might vary for men, women, and gender diverse individuals. Most importantly, they should pay attention to the significantly higher likelood that adults who identify as a sexuality or gender minority will have experienced multiple forms of child maltreatment, and make sure their identification and assessment of childhood trauma is comprehensive and attuned to this. Clinicians should also consider ways of addressing discrimination, stigma or fear based on gender and sexuality identities, which may be critical for the safety and wellbeing of their clients.

Comparison across the age cohorts in the ACMS also shows a substantial growth over time in the proportion of the population with a diverse identity. Service providers need to acknowledge and plan for the increased likelihood when dealing with younger clients, and their parents, carer and family. There are existing resources that are useful in guiding practitioners in how to be gender- and sexuality-sensitive (e.g., Australian Institute of Families Studies, 2022). However, additional support and training as part of pre-service education programs for key professional groups (like teachers, nurses, doctors, and other allied health professionals) is needed.

### Strengths and Limitations

This first analysis of child maltreatment and epidemiological data on diverse identities in Australia has considerable strengths. It is a random sample, representative of the national population, and it uses a well-validated, behaviorally specific measure that assessed the latest conceptual models of child maltreatment and open-ended questions that allowed participants to self-identify their gender and sexuality.

However, interviewers did not explicitly ask about sex assigned at birth – only about current self-identified gender. It is possible that some people who identified as men or women may have been assigned a different sex at birth. These people were classified based on their reported gender. Therefore, we cannot be sure that the data for men and women are entirely about people who are cisgendered. This may have led to very slight underestimates of transgender people, particularly if they gave their gender as simply “male” or “female”. Future studies should include sex at birth as well as current gender identity.

Our analysis uses cross-sectional and retrospective data, which precludes determination of causality. With such stark differences in the prevalence of maltreatment between those with and without diverse genders and sexualities, researchers may wonder if these variables are causally related. Further research is needed to explore, for example, whether those children/adolescents with early indications of gender or sexuality diversity make them more vulnerable to abuse and neglect, or—conversely—does experiencing childhood abuse and/or neglect—or subsequent discrimination and victimisation in adulthood—make people more likely to identify in diverse ways either during childhood/adolescence or adulthood, or the role of family rejection of children with emerging diverse entities, or their rejection of unaccepting and rejecting family.

Finally, our analyses did not look at diversity in terms of who perpetrated the maltreatment (men vs. women; parent vs. other; and—for sexual abuse—whether it was an adult vs. a peer), or the context (institutional, familial, other). Further analyses are required that look at the contribution that child maltreatment makes to any relationship between diverse sexualities and/or gender identities and health outcomes across the lifespan (health, health-risk behaviors, and mental health) – and potential mechanisms that help explain any such relationships (including temporal sequencing, causal relationships, and other variables that might play a mediating or moderating roles).

### Conclusion

For the first time in Australia, we have comprehensive, nationally representative data on the prevalence of diversity in both gender and sexuality identities, and strong empirical evidence on the strong association for both with experiences of five separate forms of child maltreatment and multi-type maltreatment. Child maltreatment primary prevention strategies, targeted interventions and therapeutic responses for children, youth and adult survivors (including justice responses) must recognise this sexuality and gender diversity, and the needs of these important—and growing—subgroups.

## Data Availability Statement

Under a registered data management plan, final data sets will be stored on the Australian Data Archive, with details for access from 2025 made available on the ACMS Web site. Under a multi-institutional agreement, the survey instrument is the intellectual property of the research team. It will be made available through a Creative Commons licence after an embargo period.

## Notes

1. Two of the identity categories, defined by the UN, including “gender-diverse” which refers to those whose gender identity “is at odds with what is perceived as being the gender norm in a particular context at a particular point in time, including those who do not place themselves in the male/female binary”. The UN also notes that “trans” is a more specific term describing those who identify with a different sex than the one assigned to them at birth. See: https://www.ohchr.org/en/special-procedures/ie-sexual-orientation-and-gender-identity/struggle-trans-and-gender-diverse-persons. There is also a range of other identities that fall outside of the binary of male/female, including: trans femme, transmasculine, gender queer, non-binary, sister girl, brother boy, agender, and those who prefer not to have a label (ABS, 2021).

2. Eight individuals stated they didn’t know, and nine refused to state their gender. Of the eight who didn’t know, six experienced child maltreatment. Of the nine who refused, five experienced child maltreatment. Therefore we decided to include both those who refused, and those who said they didn’t know in the ‘diverse genders’ category, as we wanted our measure of gender diversity to include all participants who were not comfortable identifying with the gender binary of male or female. We repeated our analyses of prevalence of child maltreatment with and without both groups, and there were only minor differences in prevalence rates of child maltreatment.

3.
https://www.abs.gov.au/ausstats/abs@.nsf/mf/1800.0
